# A Leaky Deep Intronic Splice Variant in *CLRN1* Is Associated with Non-Syndromic Retinitis Pigmentosa

**DOI:** 10.3390/genes15111363

**Published:** 2024-10-24

**Authors:** Maria Abu Elasal, Samer Khateb, Daan M. Panneman, Susanne Roosing, Frans P. M. Cremers, Eyal Banin, Dror Sharon, Asodu Sandeep Sarma

**Affiliations:** 1 Division of Ophthalmology, Hadassah Medical Center, Faculty of Medicine, The Hebrew University of Jerusalem, Jerusalem 91120, Israel; maria.abuelasal@mail.huji.ac.il (M.A.E.); samerkhateb@gmail.com (S.K.); banine@mail.huji.ac.il (E.B.); sandeepsarma.asodu@mail.huji.ac.il (A.S.S.); 2 Department of Human Genetics, Radboud University Medical Center, 6525 Nijmegen, The Netherlands; daan.panneman@radboudumc.nl (D.M.P.); susanne.roosing@radboudumc.nl (S.R.); frans.cremers@radboudumc.nl (F.P.M.C.)

**Keywords:** *CLRN1*, deep intronic, inherited retinal diseases, pseudo-exon, retinitis pigmentosa, splicing

## Abstract

Background: Inherited retinal diseases (IRDs) are clinically complex and genetically heterogeneous visual impairment disorders with varying penetrance and severity. Disease-causing variants in at least 289 nuclear and mitochondrial genes have been implicated in their pathogenesis. Methods: Whole exome sequencing results were analyzed using established pipelines and the results were further confirmed by Sanger sequencing and minigene splicing assay. Results: Exome sequencing in a 51-year-old Ashkenazi Jewish patient with non-syndromic retinitis pigmentosa (RP) identified compound heterozygous variants in the *CLRN1* gene: a known pathogenic missense [p.(N48K)] and a novel deep intronic variant c.254-643G>T. A minigene splicing assay that was performed aiming to study the effect of the c.254-643G>T variant on *CLRN1* pre-mRNA splicing revealed the inclusion of a pseudo-exon that was also reported to be included in the transcript due to an adjacent variant, c.254-649T>G. However, unlike the reported c.254-649T>G variant, c.254-643G>T showed aberrant splicing in a leaky manner, implying that the identified variant is not totally penetrant. Conclusion: We report on a novel deep intronic variant in *CLRN1* causing non-syndromic RP. The non-syndromic phenotype observed in this index case may be attributed to the leaky nature of this variant, which is causing some normal transcripts to be produced.

## 1. Introduction

Inherited retinal diseases (IRDs) encompass a large group of retinal phenotypes characterized by extensive clinical variability and large genetic heterogeneity [1,2]. Retinitis pigmentosa (RP) is the most prevalent and heterogeneous IRD and can be inherited in autosomal dominant (AD), autosomal recessive (AR), digenic, mitochondrial and X-linked (XL) modes [3].

Although it is accepted that most IRD-associated genes have been identified [4], next-generation sequencing (NGS) used as gene panels, whole exome sequencing (WES) or whole genome sequencing (WGS) fail to identify the causative genes in about 30% of IRD cases [5,6]. Although genes currently not associated with IRDs, and therefore not well covered in such analyses, might cause a fraction of these cases, elusive variants might play an important role in such cases. One example might be deep intronic variants that affect splicing, usually by introducing pseudo-exons into the transcripts [7,8,9]. A second possibility might be novel variants in genes that are associated with a similar, but not identical, phenotype. For example, genes in which all, or the vast majority, of pathogenic variants cause a syndromic multiorgan phenotype, might not always serve as candidates for the isolated non-syndromic phenotypes. Usher syndrome (RP and sensorineural hearing loss- SNHL), for example, is caused by disease-causing variants in 15 genes (https://retnet.org/; accessed on 12 August 2024), while variants in some of these genes can cause either non-syndromic RP or non-syndromic SNHL [10,11,12]. For some of these genes, this phenomenon is well established with a high number of variants causing either the syndromic or the non-syndromic phenotypes (e.g., *USH2A*), while for other genes, e.g., *USH1C*, all variants but one cause the *USH1*, while a single variant in an alternative exon causes RP with late-onset hearing loss [13]. The combination of elusive variants and genes causing multiple phenotypes is therefore challenging and requires in some cases the development of functional assays to support the pathogenicity of such variants.

The *CLRN1* gene has been identified in 2001 as the causative USH3A gene [14,15]. *CLRN1* can produce 11 transcripts through various mechanisms [15,16], and therefore, exon numbering might vary between studies. The gene, originally named USH3A, was found to have five exons and two splice variants: the main form included exons 1, 2, 3 (3a), and 4 (3b) with a 120-aa open reading frame (ORF), while the second variant included exon 1b, resulting in a 30-aa ORF. Subsequent studies refined it to CLRN1, revealing that the initial four-exon form was a rare splice variant. The main splice form consists of exons 0, 2, and 3 (3a extending into the intron between 3a and 3b), with a 232-aa ORF. All known mutations causing USH3 are located within this three-exon variant.

In the current study, we report such an elusive, deep intronic variant in the *CLRN1* gene, variants which are known to cause mainly USH type 3. This variant was found in compound heterozygosity with a well-established *CLRN1* pathogenic variant that causes USH3. The intronic variant introduces a pseudo-exon in a leaky fashion, allowing normal hearing in a 51-year-old (YO) patient presented with non-syndromic retinitis pigmentosa.

## 2. Materials and Methods

### 2.1. Patient Recruitment

Blood samples were collected from the index cases and unaffected family members. The sample collection process adhered to ethical standards, receiving approval from the Hadassah Hospital Institutional Review Board. Informed consent, obtained in writing, ensured compliance with all relevant ethical regulations and safeguarded the rights and privacy of participants. Genomic DNA extraction was carried out from blood samples using the Promega kit and Promega Maxwell (Madison, WI, USA) DNA extraction system.

### 2.2. Sanger and Next-Generation Sequencing (NGS)

WES was performed by Pronto Diagnostic (Tel-Aviv, Israel), and an IRD gene panel including 113 genes that cause RP and Leber congenital amaurosis was performed using the smMIPs platform [17]. Fastq files were uploaded to the Genoox pipeline and analyzed using Franklin (https://franklin.genoox.com/; accessed on 23 June 2020) as previously reported [6]. For segregation analysis, primers were designed using primer3 web (https://primer3.ut.ee/; accessed on 3 May 2024)), For: TCTAATGGTCTGTCTTCTCCCA; Rev: AGCCTTTAATGACCTTTCTCGG. Both variants were verified by Sanger sequencing.

### 2.3. In Silico Splicing Analysis

The effect of the deep intronic c.254-643G>T *CLRN1* (NM_174878.2) variant on its pre-mRNA splicing was predicted using Alternative Splice Site Predictor (ASSP), Berkeley Drosophila Genome Project (https://www.fruitfly.org/seq_tools/splice.html; accessed on 3 June 2024)) and the SpliceAI prediction tool.

### 2.4. Cloning

A minigene exontrap plasmid (pET01) was purchased from MoBiTec (Eupen, Belgium). A 792 bp *CLRN1* intron 1 sequence flanking the c.254-643G>T variant was PCR amplified from the genomic DNA of the patient and a control individual using PCR Taq Mix Red (PB045625-071-0, London, UK). In total, 1 µg of each PCR product and 1 µg of the pET01 plasmid were digested with *Bam*HI (1 µL) and *Xho*I (1 µL) restriction enzymes for 2 h at 37 °C. Digested products were separated on 1% agarose gel and purified using a Qiagen gel extraction kit (Hilden, Germany) (Cat. no: 28706 × 4). The DNA concentration of the gel purified PCR products and the plasmid were measured using nanodrop. The mass of the insert required for ligation was calculated using the NEBio ligation calculator. For ligation, the insert and the plasmid were mixed in a 3:1 ratio and ligated using T4 DNA ligase (NEB, Cat. no: M0202S) for 2 h at room temperature. In total, 50 µL of DH5 α-competent cells were added to each ligation mixture and incubated for 30 min on ice. After the incubation, the bacteria were heat shocked at 42c for 90 s. After heat shock, 1 ml of LB media was added to bacteria and grown for 45 min at 37c (NEB, Cat. no: C2987H). In total, 100–2001 µL of the transformed DH5 α-competent cells was plated on LB agar plates with ampicillin for the selectivity of transfected cells. Positive clones were identified using colony PCR and confirmed by Sanger sequencing. The primer sequence used for cloning is as follows: F-BamHI-CTCGAG-AGAGTAGCCTGGACTTCTTGG; XhoI-R-GGATCC-TCTGGGGATATCTGGGGAGT.

### 2.5. Transfection, RNA Isolation and cDNA Synthesis

For splicing analysis, HeLa cells were seeded into a 24-well plate and cultured until they reached 70% confluence. Cells were transfected separately with empty pET01 plasmid, wild-type and mutant *CLRN1* plasmids using (TransfeX™ Transfection Reagent, ACS-4005^™^, ATCC, Manassas, VA, USA) transfection reagent. Cells were harvested 48 h post-transfection, and the total RNA was isolated using (Quick-RNA MiniPrep, R1055, ZYMO RESEARCH, Tustin, CA, USA) by following the manufacturer’s protocol. cDNA synthesis was carried out using a qScript cDNA Synthesis Kit (Cat. No: 733-1178).

### 2.6. Automated Electrophoresis—TapeStation

Quality control of the RT-PCR products was assessed using the Agilent Technologies D1000 ScreenTape system kit and Agilent Technologies 4200 TapeStation instrument (Agilent Technologies, Waldron, Germany). After automated electrophoresis, the results were analyzed on the TapeStation Controller Software version 5.1 to detect product size and quantity with a sensitivity of 0.1 ng/µL and sizing accuracy of ±10%. The primer sequence used for PCR amplification and Sanger sequencing of *CLRN1* cDNA analysis was F-GGATTCTTCTACACACCC; R-AGGTGGGTCGAGGTCAAC.

## 3. Results

### 3.1. Clinical Analysis

**Figure 1:**
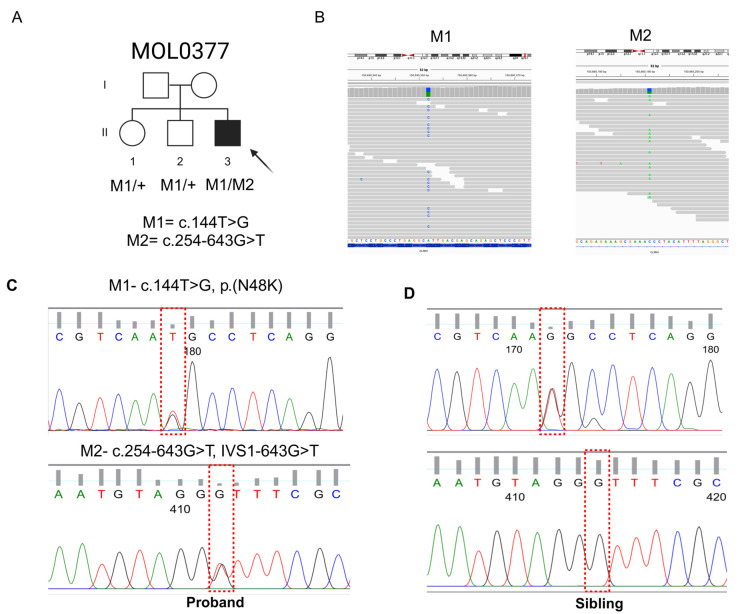
Variant detection and familial segregation analysis. (**A**) Two-generation family pedigree; (**B**) BAM files showing the two *CLRN1* variants: c.144T>G and c.254-643G>T; (**C**,**D**) Familial segregation analysis in proband (**C**) and one of his unaffected siblings (**D**). Dotted red lines shows the identified variants.

MOL0377-1 is a 51-YO male descending from a non-consanguineous Ashkenazi family (Figure 1A).

**Figure 2:**
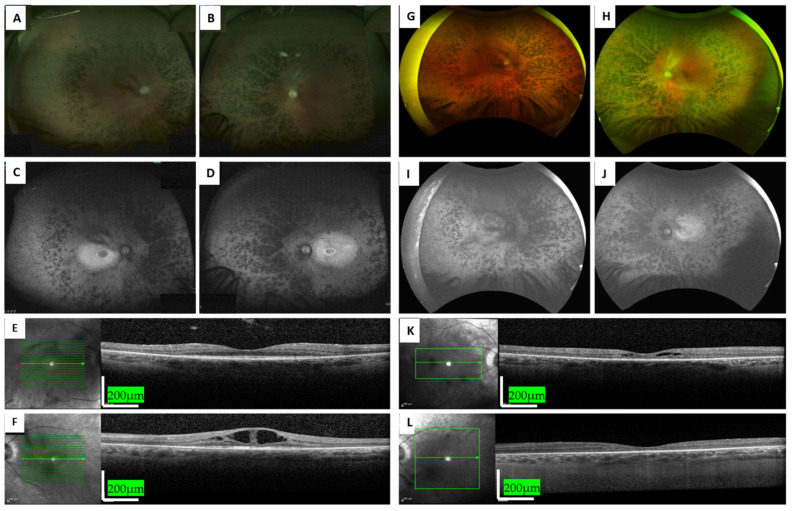
Retinal imaging of MOL377-1 at the age of 40 (**A**–**F**) and 50 (**G**–**L**). A-B and G-H represent ultra-wide-field pseudocolor and autofluorescence (FAF) fundus photos, respectively, taken using the Optos Panoramic 200 Optomap Fundus Camera. Characteristic peripheral dense BSPs mixed with retinal atrophy encroaching the temporal vascular arcades can be seen. (**C**,**D)** and (**I**,**J**) show heterogeneous autofluorescence compatible with the atrophic retina along with the hyperfluorescent ring surrounding the fovea. (**E**,**F**,**K**,**L**) are horizontal optical coherence tomography (OCT) sections showing preserved foveal islands surrounded by retinal thinning and loss of the outer retinal layers in the macular area. Cystoid macular edema (CME) was observed in the LE and RE in the first and the last follow-up visits, respectively.

At 37 YO, he was referred to our retina clinic due to complaints of blurred vision and constricted visual fields since the age of 16, which led to a diagnosis of RP at the age of 25. The best-corrected visual acuity (BCVA) at presentation was 0.80 in the right eye (RE) and 0.40 in the left eye (LE). Quiet anterior segments with mild central posterior subcapsular cataract and in fundoscopy diffuse retinal atrophy and coalescing bone specula-like pigmentations (BSPs) encroaching the macula along with narrow vessels and waxy-appearance optic discs were seen. Optical coherence tomography (OCT) showed an atrophic and dry macula in the RE, while cystoid macular edema (CME) was present in the LE (Figure 2).

Full-field electroretinography (ffERG) at the age of 25 showed extinguished rod responses and reduced cone responses, compatible with RP, and electrooculography (EOG) responses were moderately reduced. On Goldmann kinetic perimetry testing, 4IIIE target, preservation of the central 70 degrees in BE was seen. Farnsworth–Munsell D-15 and Ishihara tests were normal, and he was treated for the CME in the LE (PO Diamox 125 mg × 3). He was also treated in BE with Brominidine Tartrate (Alphagan 1 × 3/d) following his previous participation in a study examining the neuroprotective characteristics of α agonist inhibitors. He has had follow-ups for 15 years, and in the last follow-up, his BCVA was 0.32 in the BE. Of note, he underwent cataract surgery at the age of 50 in BE, including the implantation of an artificial intraocular lens. On fundoscopy, classic RP findings were observed including retinal atrophy mixed with dense BSPs, attenuated retinal vessels, and pale optic discs in BE (Figure 2). Throughout the follow-up period, he was treated with different regimens with Dorzolamide (Trusopt 2%) and Nepafenac (Nevanac 0.1%) drops for alternating CME in BE. Fundus autofluorescence images demonstrated a hyperfluorescent retina encroaching on a hyperfluorescent ring surrounding the fovea. Repeated Farnsworth–Munsell D-15 tests revealed a tendency toward the tritanopic axis. Recent Goldmann kinetic perimetry testing, 4VE target, showed preservation of the central 15–20 degrees in BE. Of note, following the genetic results (see below), the patient underwent audiometry which revealed slightly reduced responses in both ears at high frequencies which were attributed to aging and environmental exposure rather than sensorineural causes.

### 3.2. Variants Identification

**Figure 3:**
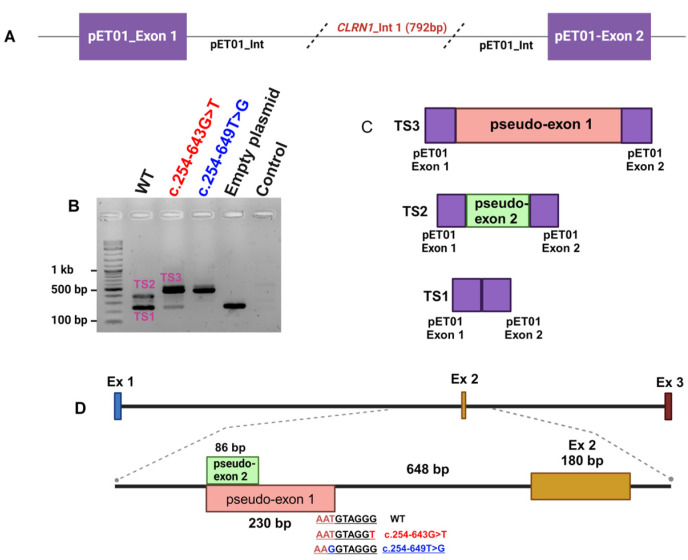
Analyzing the effect of two *CLRN1* variants (c.254-643G>T and c.254-649T>G) on its pre-mRNA splicing. (**A**) Graphical representation of pET01 minigene plasmid with a 792 bp *CLRN1* intron 1 insert. (**B**) A representative agarose gel image of cDNA analysis from HeLa cells transfected with different plasmid constructs. A 1.5% agarose gel was used to separate the PCR products; the experiment was performed in biological triplicates. Bands labeled as TS1-3 represent the different transcripts shown in panel (**C**). (**C**) Graphical representation of the different splicing patterns in the pET01-*CLRN1* minigene due to the two *CLRN1* variants c.254-643G>T and c.254-649T>G. (**D**) A graphical representation of the human *CLRN1* gene (top panel) and the insertion of a pseudo-exon due to the c.254-643G>T and c.254-649T>G variants (bottom panel) is labelled in orange color, with the sequences flanking the activated cryptic splice site. The wild-type flanking sequence is labelled in black; the previously reported c.254-649T>G variant is labelled in blue; the variant reported in the current study, c.254-643G>T, is labelled in red. A naturally occurring 83 bp CLRN1 pseudo-exon is labelled in green. Int—intron; TS—transcript; Ex—exon; WT—wild-type.

WES analysis of MOL0377-1 revealed 124,202 variants, 10,935 of which had MAF of less than 1%. Only two clear-cut reported pathogenic variants were identified in this case; both were identified as heterozygous in AR genes: *FTCD*: c.1366dupG, which has been reported to cause Formiminoglutamic Aciduria and is non-relevant to the case phenotype, and *CLRN1*: c.144T>G [p.(N48K); Figure 1], which is well described in the literature as a founder mutation in the Ashkenazi population known to cause Usher type 3 [18,19]. Three additional *CLRN1* heterozygous variants were identified by WES and smMIPs in MOL0377-1, with none of them within the open reading frame, and two of the variants showed high MAF of over 20%, excluding them as possible pathogenic variants. The remaining variant was deep intronic, c.254-643G>T (Figure 1B), identified in intron 1. This variant is extremely rare and was not found in gnomAD or other genetic databases. The variant shows a moderate score of 0.49 as splice-altering by the SpliceAI tool. Interestingly, a nearby variant, c.254-649T>G (Figure 3), with a strong SpliceAI score of 0.85 as splice-altering, was previously reported to generate a pseudo-exon [20] in a homozygous state in patients with Usher syndrome type 1. We therefore suspected that the variant we identified, c.254-643G>T, might also affect splicing. Both *CLRN1* variants identified in MOL0377-1 were verified by Sanger sequencing (Figure 1C,D). In addition, two unaffected brothers of the index case were found by Sanger sequencing to carry heterozygous missense variant, c.144T>G [p.(N48K)], but not the deep intronic variant, indicating a compound heterozygous state in the index case (Figure 1). The DNA samples of the proband’s parents are not available for segregation analysis. It appears that the proband and both of his unaffected siblings inherited the c.144T>G variant from one parent, while the second deep intronic variant in the proband may have come from the other parent. However, the possibility that this variant arose de novo in the proband cannot be ruled out. In addition, we used Sanger sequencing to screen for the deep intronic variants in 143 ethnicity-matched index cases with IRDs, none of whom was found to carry it.

### 3.3. Splicing Assay

**Figure 4:**
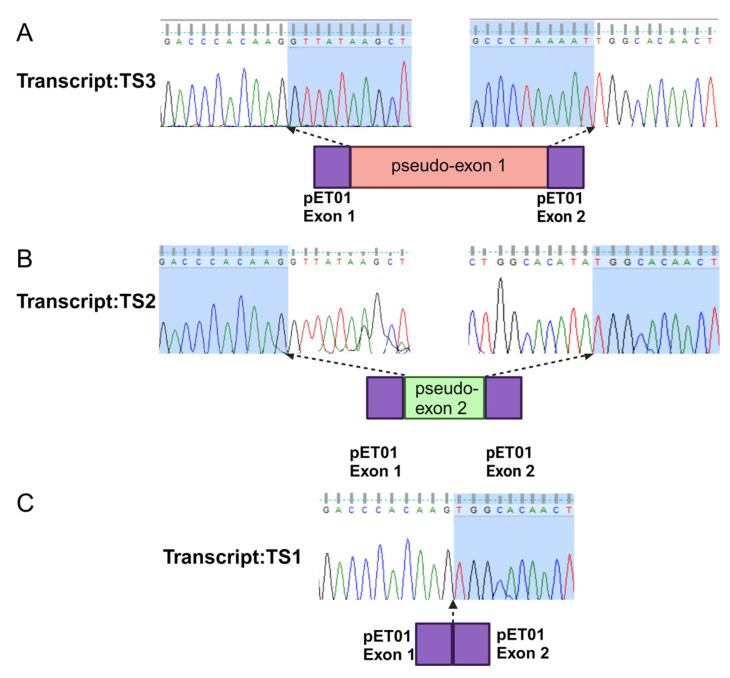
Sanger sequencing of the wild-type and mutant *CLRN1* transcripts. (**A**) Sanger sequencing of transcript TS3 containing pseudo-exon 1; (**B**) Sanger sequencing of transcript TS2 containing pseudo-exon 2; (**C**) Sanger sequencing of transcript TS1 (wild-type transcript). Light blue shadows indicate the exon-exon junctions.

**Figure 5:**
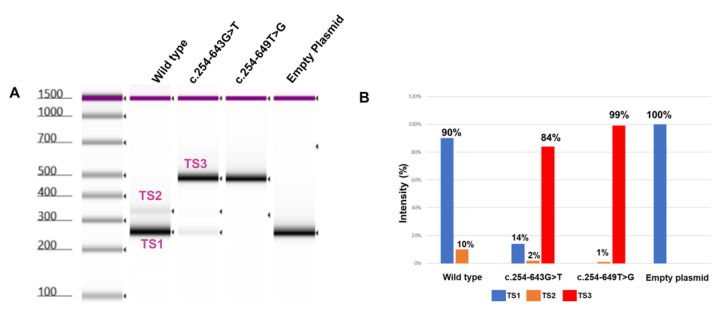
Detailed analysis of *CLRN1* transcripts using TapeStation. (**A**) Automated gel electrophoresis of the PCR products using Agilent Technologies 4200 TapeStation. The left lane is a DNA marker. “Wild type” shows transcripts produced by the normal *CLRN1* minigene, a high-intensity wild-type TS1 transcript and a very low-intensity TS2 transcript. “c.254-643G>T” shows transcripts produced by the *CLRN1* minigene carrying the 254-643G>T variant, a high-intensity TS3 transcript and a low-intensity wild-type TS1 transcript. c.254-649T>G shows transcripts produced by the *CLRN1* minigene carrying the c.254-649T>G variant, a high-intensity TS3 transcript alone. “Empty plasmid” shows transcripts produced by the pET01 empty plasmid, a high-intensity TS1 transcript alone. (**B**) Bar graph showing the intensity of each band in WT compared to the two studied variants.

To investigate the impact of the c.254-643G>T deep intronic variant on the pre-mRNA splicing, we designed an in vitro minigene splicing assay (Figure 3A). To this end, we cloned a 792 bp fragment of intron 1 flanking the c.254-643G>T variant. Three clones were generated including the wild-type (WT) sequence, c.254-643G>T, or c.254-649T>G, bearing fragments inserted into the pET01 exontrap plasmid. All three clones were validated through Sanger sequencing. The plasmids were transfected separately into HeLa cells followed by RNA isolation 48 h post-transfection. RT-PCR analysis in combination with Sanger sequencing (Figure 4) and TapeStation (Figure 5) analysis revealed a single 260 bp transcript (TS1) in cells transfected with an empty plasmid (Figure 3B), indicating the normal splicing of the pET01 plasmid minigene.

In the WT cDNA, we observed two different bands (Figure 3B): a higher-intensity TS1 band and a lower-intensity 346 bp (TS2) band indicating the inclusion of an 86 bp pseudo-exon in TS2 (Figure 4). On the other hand, in mutant (c.254-643G>T) cDNA, we observed three different transcripts: a high-intensity 490 bp (TS3) band, and faint TS1 and TS2 bands. The 490 bp band (Figure 3) represents an abnormal transcript (TS3) that includes pseudo-exon 1 (an addition of 230 bp). However, the presence of the other two splice variants (TS1 and TS2) suggests that c.254-643G>T is leaky and not fully penetrant, and some normal transcripts were also produced. On the other hand, the reported c.254-649T>G variant resulted in the production of the same pseudo-exon (#1) in a fully penetrant way, with no production of the WT transcript (Figure 3 and Figure 5). A more detailed analysis of the PCR products using TapeStation (Figure 5A) revealed similar results. We observed a high-intensity (99%) TS3 transcript in c.254-649T>G cDNA with very low to undetectable levels (1%) of TS2 and a complete absence of TS1. Similarly, we observed a high-intensity TS3 (84%) and 14% of TS1 in 254-643G>T cDNA (Figure 5B).

## 4. Discussion

Following the identification of *CLRN1* as the causative USH3A gene in 2001 [14,15], many *CLRN1* disease-causing variants have been reported. *CLRN1* can produce 11 transcripts through various mechanisms [15,16], and therefore, exon numbering might vary between studies. The LOVD-*CLRN1* database (https://databases.lovd.nl/shared/genes/CLRN1; accessed on 15 June 2024) includes 34 LP/P variants, with c.528T>G [p.(Y176*)] being the most common one identified on 44% of the mutated alleles, followed by the c.144T>G [p.(N48K)] missense variants identified on 23% of alleles. The former is relatively common in Europe (MAF of 0.005% in the gnomAD database) and especially among Finnish (MAF of 0.6%) [21]. The latter is a founder mutation in the Ashkenazi Jewish population with an MAF of 0.6%. The vast majority of *CLRN1* variants cause USH3, with a variable age of hearing loss onset and a progressive nature [22]. The average age of hearing loss diagnosis in *USH3A* patients is 8–10 years. However, in rare cases, patients who harbor relatively mild missense variants might not be aware of their hearing conditions even until the fourth decade of life or might still have normal hearing function. Therefore, some CLRN1 patients might initially be diagnosed with non-syndromic RP, and only later in life, when hearing loss becomes prominent, the diagnosis is revised to USH3. For example, two cases who were homozygous for c.606T>G [p.(N202K)] were aware of their hearing loss condition only at the age of 40 [23]. In addition, two missense variants [p.(P31L) and p.(L154W)] were reported to cause non-syndromic RP with normal audiometric assessment in two Pakistani families [24].

The deep intronic variant we identified is situated within intron 1 downstream to an intronic sequence that can potentially serve as a splice acceptor site (c.254-879_880). A previous study identified a homozygous founder mutation in Saudi Arabian patients, c.254-649T>G, which creates an intronic splice donor site, generating a 230 bp pseudo-exon within intron 1 in 87% of PCR products, with the variant located at the last nucleotide of this pseudo-exon [20]. The variant we identified introduces the same pseudogene, but based on our splicing assay, studying both variants in the same experimental setup, the variant we identified is leaky, resulting in the production of normal protein, with levels that might be sufficient for normal hearing but are not enough to protect the retina from the RP phenotype. It is essential to recognize that all the splice assays discussed were performed in cell lines that might not accurately reflect the splicing events happening in photoreceptor cells. This indicates that we could see different splicing patterns in the retina. Several earlier studies have documented mutations that exhibited varying splicing patterns in patient-derived fibroblast cells and photoreceptor precursor cells.

While patients who were homozygous for the c.254-649T>G variant were reported to have a severe USH type1 phenotype with early-onset hearing loss, the index case we are reporting here, who has a compound heterozygous for p.(N48K) (known to cause USH3 in homozygous and compound heterozygous states) and c.254-643G>T, has non-syndromic RP at the age of 51 years. Our results therefore support those reported by others that relatively mild *CLRN1* variants can cause non-syndromic RP or RP with late-onset hearing loss. We cannot rule out the possibility that the variant we identified, c.254-643G>T, might cause USH3 when in trans with a null USH3A mutation. Therefore, both USH and non-syndromic RP cases should be screened for *CLRN1* variants, not only within the ORF but also in intronic regions that might result in the inclusion of pseudo-exons when mutated. Although the two intron 1 variants we studied here seem to be extremely rare in human populations, this genomic region is susceptible to creating pseudo-exons, and therefore, sequence analysis of this region is particularly recommended, especially in IRD cases with a single identified heterozygous mutation in *CLRN1*. Such deep intronic variants might be corrected using either CRISPR/Cas9 or antisense oligonucleotides (ASOs), which were found to be efficient for correcting the positive results [25]. Both approaches can potentially rescue c.254-643G>T, which we are reporting here. Therefore, an ASO that is either located with the pseudo-exon or on the intron–pseudo-exon junction might interfere with these deep intronic variants, rescuing the CLRN1 transcripts from degradation. Alternatively, gene augmentation therapy might also rescue the phenotype caused by these deep intronic variants.

## Data Availability

Data from this study are presented within the article. The authors are willing to share materials, data sets, and protocols used in the acquisition of data presented in this publication with other researchers upon request (contact Dror Sharon, E-mail: dror.sharon1@mail.huji.ac.il).
